# How Does Confucianism Influence Health Behaviors, Health Outcomes and Medical Decisions? A Scoping Review

**DOI:** 10.1007/s10943-022-01506-8

**Published:** 2022-02-10

**Authors:** Barbara Badanta, María González-Cano-Caballero, Paola Suárez-Reina, Giancarlo Lucchetti, Rocío de Diego-Cordero

**Affiliations:** 1 Department of Nursing, Faculty of Nursing, Physiotherapy and Podiatry, University of Seville, C/Avenzoar, 6, Seville, Spain; 2 Faculty of Nursing, Physiotherapy and Podiatry, University of Seville, Seville, Spain; 3 Department of Medicine, School of Medicine, Federal University of Juiz de Fora, Juiz de Fora, 36036-900 Brazil

**Keywords:** Confucianism, Health behaviors, Medical decision-making, Transcultural nursing, Spirituality

## Abstract

The aim of this study is to analyze the influence of Confucianism on health behaviors, health outcomes and medical decisions. The research was conducted using the following databases: PubMed, Scopus, CINHAL, PsycINFO and Web of Science, without restrictions of language and year of publication. The search process identified 833 publications matching the search criteria, and after the review process, 40 articles were included. Family is a central aspect of Confucianism, and it seems to affect participation in medical decisions, taking care of relatives, ethical dilemmas and mental health problems. Although most Confucianist influence seems to be positive, some ways of thinking could increase suffering, burnout and a delay in healthcare seeking. Understanding these values could help health professionals to deal with the growing contingent of patients with different cultures and religious beliefs.

## Introduction

Interest in the study of religion/spirituality (R/S) and health has increased in the last decades (Lucchetti et al., 2016) generally supporting a positive association between R/S and health outcomes (Akerman et al., 2020). Some of the explanations for these findings are related to the beliefs of religious traditions which tend to avoid harmful behaviors and promote social connection and attendance to certain rituals that have beneficial effects on physical and mental health (Chen & Vanderweele, 2018). However, the training offered in universities and centers is scarce, reducing the ability of students and healthcare professionals to offer it in a correct way (de Diego Cordero et al., 2018, 2019).

Despite the evidence, the differences and similarities of religion and spirituality are not totally clear. Religion is defined as the set of practices, beliefs and rituals related to the existence of a superior being or God to whom devotion is paid and responsibility for certain events is attributed (Forti et al., 2020). However, if religion is defined according to the Judeo-Christian model, it might be justified in saying that Confucianism is not a religion (Adler, 2011). In this case, spirituality appears as a broader concept, going beyond religious values and doctrines (Sessanna et al., 2007), where people initiate the search for the meaning and purpose of life which may or may not be related to religion (Koenig et al., 2001). Using this definitions, a person could be spiritual but not religious, which is very common in Eastern cultures and traditions, such as in Confucianism.

### Pillars of Confucian Thinking

Confucianism is based on the principles of the good life, loyalty and respect for older persons and family, as well as encouraging harmony and altruism (Chen, 2001). These Confucian ideas have been passed down through many dynasties, and, despite the rejection it received in the 1970s, it is now widely accepted in Eastern culture. It can be explained because the Confucianism may be considered a safeguard to find the meaning of life in secular societies (Shek et al., 2013).

The pillars of Confucianism are composed by twelve basic virtues which are detailed as follows: *Zhong* (loyalty) is related to the fulfillment of duty and the utmost commitment to it, coupled with impartiality in decision-making; *Xiao* (filial piety) is the respect and sense of obligation to parents, fulfilling their will and beliefs until the last moment; *Ren* (benevolence) is the respect for one's own life and that of others contributing to generosity and humility, which can bring about positive political and social changes; *Ai* (affection) is the basis for being able to love, as it implies the care of interpersonal relationship centered on respect and humanity; *Xin* (trustworthiness) encourage the honesty and trust in other people; *Yi* (righteousness) is not falling into temptation to follow the right path in fulfilling orders; *He* (harmony) includes the participation of people for the transformation and achievement of social order and *Ping* (peace) refers to peace of mind and calmness; all, as a whole, make up the eight cardinal virtues. The rest are *Li* (propriety) through guidelines and models for human relationships and social order; *Zhin* (wisdom) in reference to morality through reflection, imitation and experience; *Lian* (integrity) as moderation to avoid behaviors that lead to partiality and ruin; and finally *Chi* (humility/shame) experienced by incorrect behaviors (Shek et al., 2013).

### Health in the Confucian Context

Confucian teachings are mainly based on the virtue of benevolence based on filial piety and fraternal submission (Koh & Koh, 2008), highlighting the need to know the Chinese family structure in order to provide quality care. Thus, the family is the main channel for decisions related to health, as well as the source of economic resources to face the health care of any of its members (Cao et al., 2011).

The societies that follow Confucianism function through defined hierarchies where different roles are established with their respective obligations and duties, which must be fulfilled to achieve personal harmony, while rejecting the pursuit of personal happiness originated by desires and impulses. This leads to the idea that mental health problems could be the result of lack of self-discipline and weakness of character (Huang & Charter, 1996).

Several studies have investigated the differences of beliefs of Confucianism in relation to other Western traditions. As an example, women have a pivotal role as primary caregivers for children with Down Syndrome, being responsible for maintaining the balance of the whole family in this situation (Choi & Van Riper, 2017). In relation to adherence to treatment, Chinese individuals have different postures as compared to Western individuals, accepting health recommendations unquestioningly and waiting for the health professional to finish the explanation without interrupting (Choi et al., 2017). Beliefs also have an important influence on ethical and critical situations, such as the rejection of the concept of "brain death", since Confucianist individuals do not understand the dichotomy of soul and body and, the moment the body granted by the parents, is eliminated, they and their ancestors are desecrated (Yang & Miller, 2015).

Since the Eastern societies and migration are increasing, addressing and understanding these cultural differences are important to health managers and healthcare professionals. Nevertheless, there has been little evidence compiling articles on the relationship between Confucianism and health behaviors and medical decisions and, to our knowledge, no scoping review. Our aim is, therefore, to assess through a scoping review the influence of Confucianism on the provision of health services, health behaviors and medical decisions.

## Methods

A scoping review was carried out following the framework set out by Arksey and O’Malley (2005) and expanded upon by Daudt et al., (2013). Five stages were followed: (1) identifying the research question; (2) identifying relevant studies; (3) study selection; (4) charting the data; and (5) collating, summarizing and reporting the results. Then, a critical review to summarize the ideas and information was done. Authors have assessed carefully and clearly each reference and have took into consideration both the strengths and weaknesses of the articles under review.

### Search Methods and Strategy

This scoping review has been registered on PROSPERO under ID CRD42021248022 and has followed the PRISMA guidelines. The search was conducted independently by two researchers between January and March 2021 in five electronic databases (PubMed, Scopus, CINHAL, PsycINFO and Web of Science). The following search strategy was used: (confucianism OR confucian) AND (health OR “health promotion” OR lifestyles OR “healthy lifestyle” OR “health behaviors”).

The search strategy was elaborated following the PICOTS structure as visualized in Table 1.

**Table 1:** PICOTS (population, intervention/exposure, comparator, outcome, time and study design) criteria

PICOTS criteria	
Population	Confucian population
Intervention/Exposure	Confucian principles or values related to health
Comparator	Western intervention/values, standard practice, no comparator
Outcome	Confucianism as modulator of the provision of health services, health behaviors and medical decisions
Time	Not applicable
Study design	Cross-sectional, case–control, longitudinal cohort and descriptive, ecological or intervention studies, natural experiments, and literature reviews

### Inclusion and Exclusion Criteria

To be included, articles should be literature reviews or original articles using the following designs: cross-sectional, case–control, longitudinal cohort, descriptive, ecological or intervention studies and natural experiments.

There were no restrictions on language and year of publication. Exclusion criteria were published materials such as conference abstracts, case reports, editorials, letters to the editor and book chapters. Articles that related Confucianism with non-health outcomes (e.g., economics, politics) were also excluded, or those where the relationship between Confucianism and health was not the central focus of the results (e.g., theoretical essays or reflections).

### Selection of Studies and Analysis

The first phase of the review was carried out by two researchers who independently assessed the titles and abstracts of all references. Duplicate articles were removed manually. A third researcher was in charge of reviewing the articles with discrepancies and including it or not. Included articles were then moved to the next phase.

In a second phase, data extraction and data verification were carried out by two researchers independently who assessed the full-text articles. The authors classified and then ranked the studies as being (i) poor, (ii) good or (iii) very good based on their strengths and errors detected mainly in the methodology, presentation of their results and discussion. All studies were classified as good or very good and all authors had to agree. The eligible articles are then included in Table 3, which contained reference/context, purpose of study, design method, measure(s), sample and major findings.

## Results

**Fig. 1:**
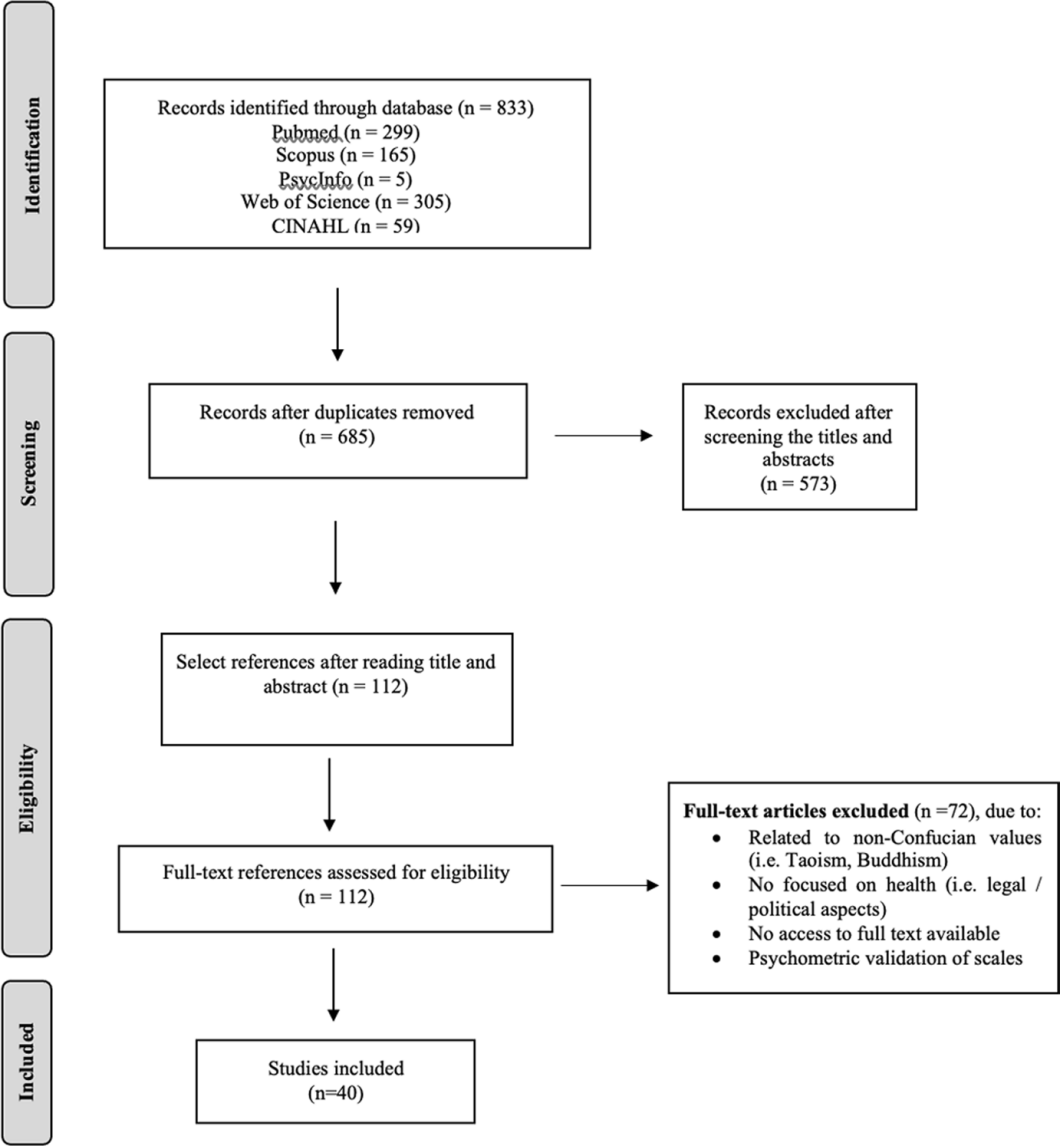
Flowchart for the selection of articles for the scoping review

The search process identified 833 publications matching the search criteria (Fig. 1). After removing duplicates, 685 articles remained, of which another 573 articles were excluded after screening the titles and abstracts. A total of 112 articles underwent full-text analysis. After reading the full text of the articles, the final sample included 40 studies: 13 using qualitative design, 13 quantitative studies, 9 narrative reviews, 4 essays and 1 systematic review.

### Characteristics of the Included Studies

Forty articles addressing the influence of Confucianism on the provision of health care, health behaviors and medical decisions were included in this review. More than half of the references were published before 2017. Studies carried out in Eastern countries predominate (*n* = 32; 80%) and seven articles included immigrant population living in Western countries. The quantitative studies were predominantly cross-sectional, and there was only one clinical trial. All qualitative methodology articles used interviews for data collection.

Most participants included in the studies were females and the age of the participants ranged between 15 and 92 years. Four studies included only samples of women, and in eleven publications, their representation ranged between 50 and 80%. Familism, filial piety, harmony and benevolence were the main Confucian values included in the studies as shown in Table 2.

**Table 2:** Confucian values, virtues, and beliefs related to health

Health Context	Confucian values, virtues and beliefs		References
Medical decision making	Familism, Filial piety, and Harmony	Family-based consent is autonomous consent and it enhances the solidarity of family members and throughout the larger society. Relatives have the same right to be informed and make decisions to protect the patient, so the patient makes health decision but in harmony with the family	Cong, (2004); Chen & Fan, (2010); Fan & Wang, (2015); Raposo, (2019)
		Medical decision-making is a responsibility assumed by the children, as a symbol of gratitude and respect toward their senile parents in compliance with filial piety. Older parents also feel the duty to take their children into account when making decisions	Lee et al., (2016); Tai & Tsai, (2003)
		Familism implies sacrifice and not causing problems or embarrassment to the family. Therefore, when the patient suffers from stigmatizing health problems, reporting information to the patient individually protects the family from shame and discrimination by others in the community. Patients feel that they must manage these health problems without family members or healthcare professionals	Chen et al., (2007); Kang & Crogan, (2008)
Provision of health care and Health behaviors	Familism; Filial piety, Benevolence	Family responsibility for the care of children and dependents (physical, psychological and material), instead of governments. Feeling of guilt when placing a family member to an institution Self-care so as not to burden family members and acceptance of family care as a way of guaranteeing filial piety of children Children should care for their elderly parents, thus maintaining the linear family structure	Cao et al., (2011); Chang & Basnyat, (2017); Fan, (2006); Holroy, (2001); Holroy, (2003); Laidlaw et al., (2010); Lo & Rusell, (2007(; Park, (2012); Tao et al., (2014); Yiu et al., (2021); Zhang, (2010)
		Strength of family virtue, continuity of the surname and veneration of ancestors of the family patriarch, which required male descent. Importance of the role of women in the family (nei), which can influence pregnancy behaviors	Chung, (2007)(
	Filial piety	Filial piety involves cultivating heterosexual sexual virtue and avoiding premarital sex	Feng et al., (2012); Gao et al., (2012)
	Harmony and morality	A non-harmonic situation between the person and the environment can cause a disease or symptom Respect for traditional values and greater use of cultural practices such as Traditional Chinese Medicine	Chen et al., (2008); Lai & Surood, (2009); Rochelle & Yim, (2014)
	Symbolic capital	Promotion of health practices to keep the social hierarchy of the family through the intergenerational transmission of values	Pang et al., (2015)
	Tam giao	Importance of facing chaos to achieve social harmony and promote community solidarity. Detachment of individualistic attitudes related to health management	Small & Blanc, (2021)
	Obedience to authority	Collective obedience to the government to curb infections during the COVID-19 pandemic and reduce mortality, through attention to cleanliness, maintenance of social distance and other restrictions	Mayer et al., (2020)
Dignity, Death and Body donation	Universal dignity and personal dignity	Safeguarding one’s life is at the core of preserving one’s universal dignity, although certain circumstances, one should sacrifice one’s life to preserve one’s personal dignity Filial piety mentions obeying parents, but also serving them, that is, taking care of them and keeping them alive	Li & Li, (2017)
	Filial piety Benevolence	The body, hair and skin are given by the parents, and one cannot damage them Based on filial piety, individuals' bodies are owned by the parents, and they must be intact at death. However, benevolence allows for body donation as people have a moral duty to help others. Moreover, rites as other Confucian value, indicate how body donation should be performed as a communal activity The family-oriented benevolence requires family approval for organ donation	Cai, (2019); Jones & Nie, (2018); Nie & Jones, (2019); Zhang et al., (2020)
Mental Health	Fate thinking	Predeterminated destiny: the course and culmination of human life is both transcendent and beyond human control, such as prosperities and sufferings. Confucian culture emphasize fate thinking but does not advocate inaction	Lihua et al., (2017)
	Zhongyong (the Doctrine of the Mean)	Balance and harmony from directing the mind to a state of constant equilibrium	Yang et al., (2016)
	Familism and Filial piety	Requirement to obey parents and fulfill family roles so as not to cause dishonor. Avoiding shame and maintaining family reputation can lead to an experience of isolation and discrimination of patients with mental health problems by their families and communities	Hsiao et al., (2006); Park, (2012); Ran et al., (2021)
	Work-related Confucian values (hardworking, endurance, reciprocity, and loyalty)	It favors the relations between workers and the company and their objectives	Siu, (2003)
	Hakbeol	Status in Korean culture based on the belief that socially desirable values (e.g., higher social status, wealth, power) are based on one’s educational achievements	Garrison et al., (2018)
	Filial piety and harmony	Los valores confucianos pueden afectar a las tasas de suicidios	Jia & Zhang, (2017)
	Tam Giao	Importance of facing chaos to achieve social harmony and promote community solidarity. Social solidarity acts as protective psychological factors	Small & Blanc, (2021)

### Confucian Values, Virtues and Beliefs Related to Health

**Fig. 2:**
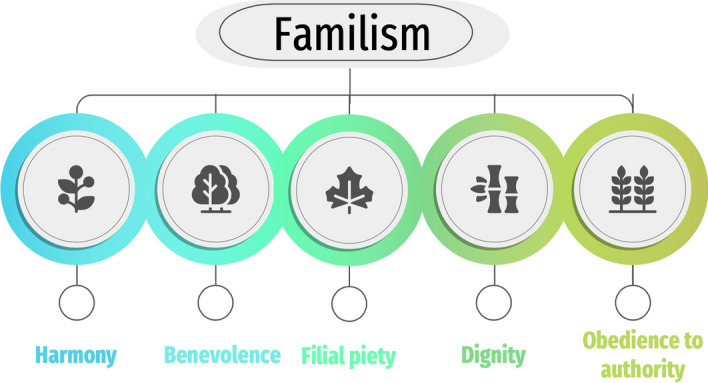
Familism as the main axis of Confucianism

Among all Confucian values, virtues and beliefs, those that place the highest value on the family stand out (Fig. 2). Familism is a Confucian value that puts family at the center of importance, and identifies that the human being is not self-sufficient for leading a good life independently from others (Chen & Fan, 2010). This implies respect to family relationships, mainly toward parents, perceived as filial piety or family reverence (Tai & Tsai, 2003), which is articulated as a starting point for the practice of benevolence (Zhang, 2010).

In this context, society is also understood as a family, which makes health behaviors linked to socially desirable values, in order to maintain dignity, social harmony and obedience (Garrison et al., 2018; Small & Blanc, 2021).s

### Family as Focus of Medical Decision-Making

In Confucianism, medical decisions are highly influenced by its values such as familism, filial piety and harmony. In clinical practice, the family plays a pivotal role in health care decisions, in a way that the family informed consent is more important than the personal informed consent (Cong, 2004). The participation of the entire family ensures good relationship and avoids unnecessary suffering to the patient, using the justification that the patients could be deprived of information or the truth about their health status (Chen & Fan, 2010). However, this does not always imply excluding the individual from decision-making, but rather inviting physicians, patients and their family members, so that relevant persons must be involved and their diverse views respected and reflected before a harmonious agreement be made (Chen & Fan, 2010).

Another important value is the filial piety when parents become senile. In this situation, the oldest son is the responsible of taking care of them, including for making decisions (Tai & Tsai, 2003). This way of acting of the children is expected by their parents and in some circumstances avoid the search for healthcare, as shown in a study among women with urinary incontinence (Kang & Crogan, 2008). In short, patients themselves consider that the head of the family should know the patient's medical diagnosis, including cancer, and make the decision to initiate treatments (Raposo, 2019), which also includes the “Do not Resuscitate” orders (Lee et al., 2016). Nevertheless, at the same time that children fulfill their obligations, parents must also develop personal morality as a mean to make family proud. Hence, they avoid discussing issues such as death and involve third parties in decision-making at the end of life, since individual acts could be interpreted as a less willingness to sacrifice (Lee et al., 2016).

On the other hand, shame or guilt can also lead to solitary decision-making and refusal of healthcare professionals seeking care. In China, the stigma and social discrimination associated with HIV disrupts the traditional Confucian practice based on the beneficence of disclosing diagnostic information to family members. In these cases, the information to the patient is consistent with Confucianism, as patients prefer to address the disease themselves to protect their family from embarrassment by other members of the community (Chen et al., 2007).

Finally, there is also an important challenge to the Confucian medical decision-making model. The influence of Western vision on health laws would leave out of place the authority that families have in making decisions about hospitalization and treatment of family members (Fan & Wang, 2015).

### Provision of Health Care and Health Behaviors

The family as the primary social unit and sacrifice is the basis of family care and functioning and, for this reason, the other family members should assume all the functions that the sick person cannot perform. This results in sacrificing employment and leisure time to have available time to take care of the relative. Confucian society accepts that care for older persons and dependents is carried out within the family, or when this is not possible, by the community or neighbors (Fan, 2006), while institutionalization is not considered an honor or the best option. Due to social changes and westernization of health, options for family–government collaboration appear, mainly of an economic nature (Cao et al., 2011; Lo and Rusell, 2007; Zhang, 2010).

According to Confucianism, acting as family caregivers is part of a “Heaven’s plan” (Yiu et al., 2021), and this type of care focuses on moral values such as embody benevolence, righteousness or justice, and propriety or courtesy (Holroyd, 2001, 2003). The Confucian principles of mutual and reciprocal obligations are cyclic and works as follows: parents provide for their children’s care and education while children practice filial piety, the responsibility to respect and care for their parents (Laidlaw et al., 2010; Park, 2012). They tend to give more importance to comply with this Confucian values than to achieve positive health results and for this reason, sacrifice of its own health in detriment of the other in need may be acceptable.

However, this review finds ambivalence between benevolence and filial piety. On the one hand, patients are pleased that their families expresses concern and love through caring. On the other hand, they stimulate self-care in an attempt to avoid being a burden to their family. In addition, self-care is practiced under the belief that positive moods can improve bodily health, hence the individual coping with suffering and pain as occurs in patients with cancer (Chen et al., 2008). Therefore, different strategies have been used to improve mental status, such as feeding livestock and poultry, planting vegetables and flowers, watching television, and attending recreational activities (Tao et al., 2014).

As part of caring, it is also important to mention that the adherence to Confucian norms are directly related to the use of traditional medicine, and healthy patterns of physical activity and eating (Lai & Surood, 2009; Rochelle & Yim, 2014). However, in Pang et al. (2015), parents conceptualized sport as valuable only if it contributed to substantial symbolic capital. Given that the fulfillment of filial piety implies respecting the health knowledge and preferences of the older persons of the family (for example, about TCM), this can sometimes be incorrect (Chang & Basnyat, 2017) or be a risk of acquisition unhealthy habits.

Traditional Confucian values also modulate sexual behaviors. Confucian women were less likely to choose an induced abortion (Chung, 2007), and young people had less premarital sex. In short, the Confucian tradition places greater emphasis on the spiritual pursuit of morality in young men, rather than affirming masculinity by overt sexual activity (Gao et al., 2012). Connecting with this, Confucianism emphasizes that people should procreate to carry on their family bloodline, so those with more traditional Confucian values have a worse perception of homosexuality (Feng et al., 2012).

Finally, the management of the COVID-19 pandemic situation is also related to positive health outcomes in these Confucian societies. Confucian values, including *Tam Giao*, tend to create a community spirit that influences high obedience rates, greater attention to cleanliness, and the promotion of collective solidarity (Mayer et al., 2020; Small & Blanc, 2021).

### Dignity, Death, and Body Donation

Confucian principles of morality and dignity encourage individuals to continue living, to fulfill their responsibilities in the family and in society. In this context, deciding individually to end life has a negative impact on family reputation and is therefore considered totally inappropriate. However, preserving the quality of life—equivalent to moral quality or dignity, which is essential to live in a Confucian society, allows individuals to decide not to deliberately extend life using technological advances (i.e., dysthanasia) (Li & Li, 2017).

Regarding studies focusing on organ donation after death, these reflect the influence of filial piety and benevolence (Cai, 2019; Zhang et al., 2020). Filial piety could get in the way of organ donation, since although it entails an act of compassion and righteousness toward others, based on this Confucian value, the basic duty of the children is keeping the body in the condition in which it was given to him/her by his/her parents. Therefore, Confucianism does not advocate body donation, justifying this posture as maintaining the love and respect for the family and the dying person (Cai, 2019; Nie and Gareth Jones, 2019).

However, values such as humaneness or benevolence comprise the body donation as virtuous acts to promote virtue cultivation and honor the relatives (Jones & Nie, 2018). In cases such as the donation of organs from prisoners or people who have committed dishonorable acts for the family, the donation could be approved by the family (Cai, 2019; Jones & Nie, 2018).

### Mental Health

Some Confucian values and principles are considered protective elements of mental health. For instance, the *Zhongyong* (“Doctrine of the Mean”—one of four Confucian texts) is suggested to maintain harmony of mind and alleviate depressive symptoms (Yang et al., 2016). Likewise, a previous study found that those with higher levels of Confucian work values were able to respond better to stressful situations at work (Siu, 2003). This was also observed in the COVID-19 pandemic where Confucian values were considered important markers to the development of resilience. As Small and Blanc (2021) point out, *Tam Giao* (which includes Buddhism, Confucianism and Taoism) may have tools to provide protective psychological factors that are derived from social solidarity, and a positive view of government actions to prevent the spread of the COVID-19 pandemic.

Despite the aforementioned positive effects, it is important to highlight that fate thinking (the core of Confucian thoughts) was also associated with higher depressive and anxiety symptoms (Lihua et al., 2017). A previous work has found that obeying to the filial piety and the harmony of Confucian doctrine increased suicide cases in women with depression (Jia & Zhang, 2017). This could be explained because Confucian interpersonal peace can be affected if culturally accepted expectations and behaviors are not met, with feelings of shame and guilt appearing, which can lead to depressive problems (Garrison et al., 2018; Hsiao et al., 2006).

Finally, in Confucianism, mental health problems are often associated with bad life habits, generating guilt and social stigma for both individuals and their families. To avoid recognizing these problems, the search for treatment is delayed and patients are isolated and admitted to health centers. This makes the family feel dishonored and guilty, and the individuals for their part, feel the failure of having raised children who do not honor filial piety (Park, 2012; Ran et al., 2021).

The summary of all studies included in this review is shown as "Appendix 1".

## Discussion

As shown in our review, Confucianism is still very common in the Eastern traditions and influences many aspects of life in countries such as China, Japan and Korea with regard to mental and physical health, quality of life and ethical situations (Abdullah & Brown, 2011; Chiu, 2001; Li & Hou, 2012). As previously shown, family is the most important aspect of Confucian values and all decisions are based on the good of the whole family, in other words, “there is no self in Confucian teachings” (Tung, 2010). In this context, the family tends to prevent the isolation and the detrimental psychological effects of cultural alienation (Lee, 2015). This is supported by the fact that this relationship is maintained even after migration to Western countries, as seen in Chinese immigrants in the USA and Canada (Tung, 2010).

Family is so important to this system of beliefs that parents take care of their children under an authoritarian parenting style, to comply with obedience to the older persons (Hu et al., 2014). Likewise, it is the responsibility of youngers to take care of the older adults. Caregivers experience pressure from the local community to keep the family together. Raising a kin's child is not only a family matter; rather' it indicates social merit and involves the risk of being judged by others (Hu et al., 2014).

Although family can be a source of union and promotion of health, the pressure of community and the detriment of own health to the health of family members could be deleterious as well. For instance, familial obligations may become first than health care and family opinions have a stronger influence over health professionals, in a way that treatments tend to start using medications provided by family members (Tung, 2010).

Ethical issues, medical decisions and end-of-life care are other important areas influenced by Confucianism. As Confucius stated, “The body and hair and skin are received from the parents, and may not be injured” (Lin, 2009: 186). This statement shows the importance of filial piety and results in several consequences for organ donation and ethical dilemmas (Cai, 2019; Zhang et al., 2020). Health professionals should be aware of this characteristic of Asian patients in order to provide an appropriate assistance.

In relation to public health implications, it seems that there is an apparent divergence between modern healthcare systems and Confucian values. Western traditions tend to be very distinct from Eastern traditions and treating Confucian immigrants is no easy task, since most of the Western systems are not prepared to address these patients, resulting in worse outcomes such as poor healthcare and conflicts (Tung, 2010). However, this divergence is now appearing even in the Asian countries such as China. The healthcare reform in China has increase the technology and costs of healthcare, moving it away from the Confucian values of benevolence, humanity and justice (Cao et al., 2008; Du, 2008). According to Cao et al. (Cao et al., 2008), it is now essential that the new Chinese healthcare system is attached to traditional Confucian cultural concepts such as “people as the foremost”, “the benevolent man loving others”, “medical treatment as the principle and humanity as the method”, and “valuing justice above material gains”. This will make this system more holistic and inclusive.

From a Western perspective, in countries with consolidated and modern healthcare systems and high number of Asian immigrants, it is essential to understand Confucian values, incorporate them as cultural competencies and train health providers to deal with their positive and negative aspects, fully integrating these immigrants.

All this aspects could be affected during the currently COVID-19 pandemic, and this pandemic shape the future of religious ethics in general and in Confucianism in particular, due to the religious, cultural, ethical, and political implications. For adherents of Confucianism only through caring for others can they possibly care for themselves, so, the life and salvation do not an individual matter but a familiar and community aspect, and this is in contrast to a general vision of society that insists on a foundational individualism or ethnocentrism in ethics and politics (Alimi et al., 2020).

## Study Limitations

The present review has some limitations that should be highlighted. Some keywords related to Confucianism such as “benevolence”, “family”, and “justice” were not used. A more broad approach could have contaminated our findings, resulting in a huge number of unrelated articles. Therefore, our approach was to include the specific term “Confucianism” and “Confucian”. Likewise, although several databases were searched, no Chinese databases were used, and it is possible that some studies have not been included. In addition, the lack of clinical trials within the articles included in our scoping review could be considered a limitation. Finally, although the authors shared strengths and errors of the articles and agreed to classify them as good or very good, no methodological quality control tool (i.e., STROBE, SRQR, and CONSORT) was used.

## Conclusions

Our review has provided further evidence regarding the influence of Confucianism on health behaviors, health outcomes and medical decisions. Based on our findings, family is the central aspect of Confucianism and it seems to affect many dimensions of life, including the participation in medical decisions, taking care of relatives, ethical dilemmas (e.g., organ donation and end-of-life issues) and mental health problems. Although most of the influence seems to be positive and lead to better outcomes, some ways of thinking could be deleterious as well, increasing suffering, burnout, and a delay in healthcare seeking. Understanding these values could help health managers and health professionals to deal with the growing contingent of patients with different views and cultures.

With regard to future implications for clinical practice, given that international migration is increasing and it will cause changes in the sociocultural demographics of many countries, it would be convenient to replicate this type of research with other religious affiliations, aiming to adapt clinical care.

## Data Availability

The data that support the findings of this study are available from the corresponding author upon reasonable request.

## Appendix 1

See Table

**Table 3:** Results of the scoping review

Reference / Context	Purpose of study	Design method and Related measure(s)	Sample	Major findings
Cai, 2019/ China	To analyze the attitude toward organ donation through the Reconstructionist Confucianism's perspective	Narrative review	–	The family-oriented benevolence requires family approval for organ donation and implies that individual consent must be rejected. Otherwise, this act would be considered inappropriate, especially if individuals focused on external goals (i.e., reputation, recognition, or the respect of others, personal benefit) rather than on the good of the family The Classic of Filial Piety in a literal manner could make organ donation impossible, since individuals cannot harm the body given by parents. However, family-based consent for organization donation is compatible with Confucianism
Cao et al., 2011 / China	To analyze the Chinese healthcare system and its emphasis on family centralization from a Confucian perspective	Essay	–	Confucian thought considers that the primary locus of financial responsibility for health care is the family, not the individual or the government. However, the current Chinese healthcare system is neither family-oriented nor financially sustainable. Health insurances are different depending on the city of residence and they are individual, which leads to a decentralized care of the whole family and a mismatch of health costs
Chang & Basnyat, 2017 / Singapore	To understand the cultural experience of older Chinese women in Singapore on support provision and receipt	Qualitative (semistructured interviews)	38 Older Chinese women Gender: 100% female Age: 60–79 years (M = 66.7)	Confucian norms expect both older and younger family members to make contributions to the family. The information provided by older people is not questioned by the participants, because the family members respect and trust them. Older people offer knowledge about TCM (diet, food, herbs, and remedies) to keep the family healthy Women maintain a cultural role as wives, mothers, support providers, and their sense of family responsibility is carried on in advanced age, prioritizing family obligations over personal health. The filial piety of the children makes them to provide physical assistance, companionship, and material aid, but only when physical and financial dependence was unavoidable for the women
Chen et al., 2007/ China	To understand HIV attention in China provided by healthcare professionals (HCP)	Qualitative (semistructured interviews)	29 HIV-positive patients Gender: 75.8% male Age: 23–72 years (M = 38)	In China, the stigma and discrimination associated with HIV (particularly when people have homosexual sex or intravenous drug use) disrupt the traditional Confucian practice based on the beneficence of disclosing diagnostic information to family members. Therefore, participants prefer that doctors inform them first about their diagnosis because they need privacy to rearrange their lives However, reporting the diagnosis individually is also consistent with Confucianism, as it protects the family from shame and discrimination by others in the community HCPs often are a buffer against stigma and a source of support for those individuals who wish to keep their serostatus secret
Chen et al., 2008 / China, USA	To explain concepts from Chinese culture that influence the oncological pain experience	Narrative review	**-**	From the Confucianism, pain is a way for showing that people are not insensitive, and humanness is to share with others one’s suffering. For Chinese patients, their cancer pain experience may be an essential part of life, and they rather endure the pain and not report it to the medical staff until it becomes unbearable
Chen & Fan, 2010 / China	To describe the Chinese family-based and harmony-oriented model of medical decision-making	Essay	**-**	Based on Confucianism, if an individual does not form a unity with another individual in an appropriate manner, the person will be unable to attain a normal human life or harmony. To guarantee harmony, patient, doctors and family are relevant in the decision-making process and all must be engaged by carefully considering others' views. Therefore, when a family member is ill, he / she should not undertake the burden of making medical decisions independently. The patient does not have an absolute right to know the truth concerning his/her medical condition. If family members judge that informing the diagnosis and/or prognosis to the patient would have a damaging psychological effect on the patient, they could ask the physician to dilute the severity of, and even hide the information from the patient
Chung, 2007/ South Korea	To know the induced abortion in a society with three distinct religions: Confucianism, Buddhism and Christianity	Quantitative (based on the 2000 Korea National Fertility and Family Health Survey)	6348 married women Gender: 100% female Age: 15–49 years	Christian women had a considerably decreased incidence of induced abortion compared with Confucian women (OR = 0.39, 95% CI = 0.18, 0.88). However, there was no significant difference in incidence of induced abortion between Buddhist women and Confucian women
Cong, 2004/ China	To explore bioethical practices on the doctor-patient/family-patient relationship (DPR and DFPR) and informed consent in China	Qualitative (interviews)	12 physicians, 3 Chinese patients and 3 patients’ relatives	Although patients give their consent and their wishes are being considered, the medical decisions are made by the whole family as co-decision-makers, with a representative family member acting as a link between the patient and the doctor The involvement of a family member is a protection for the patient, but he / she is not fully informed of the illness because of the physician-regarding limitations of disclosure. However, neither the patient nor the family member expressed dissatisfaction with the current DFPR model
Fan, 2006 / China	To describe the benefits of family and vicinity care based on Confucian moral against institutional care	Essay	–	Based on the Confucian conception of familism and filial piety, institution care is not a morally good choice, so it rejects placing the elderly in institutions. The first care option is delegated to the family and the vicinity care would be the second best Vicinity care draws on the resources of a community to care for the elderly, without separating them from adults and young people as in institution care. This model of care is necessary for children to be obedient to rites and practice virtue. In addition, Confucians understand that the mutual psychological support is crucial, and family relationships provide experiences of meaning and intimacy that cannot be replaced
Fan & Wang, 2015/ China	To present reactions within the medical community to the Chinese Mental Health Act of 2013	Essay		From the Chinese approach, family as a whole has both the authority and the moral responsibility to initiate involuntary hospitalization and treatment of a mentally ill family member. However, Chinese lawmakers influenced by Western individualistic views, did not integrate familist concerns into the Chinese Mental Health Act of 2013 Authors propose that Article 30 should specify that a patient who is not at risk of causing physical harm to self or others, may be involuntary hospitalized and treated with the consent of the major family members
Feng et al., 2012/ China, Vietnam	To know adolescents’ and young adults’ perception of homosexuality in three Confucian Asian cities	Quantitative (cross-sectional survey)	17,016 male and female, married and unmarried adolescents and young adults Gender: 50.6% male Age: 15–24 years	Adolescents and young adults who scored as more traditional on family values, gender role values, and attitude to premarital sex tend toward a more negative perception of homosexuality. Only among Hanoi females, traditional family values are significantly related to the perception homosexuality is abnormal and unacceptable. A liberal attitude toward female premarital sex was also associated with a positive attitude toward homosexuality among Hanoi and Taipei males, and both genders in Shanghai
Gao et al., 2012/ China, Vietnam	To examine how the changes in traditional Confucian values impact the sexual behaviors of adolescents and young adults	Quantitative	16,554 male and female never married, conducted in urban Hanoi, Shanghai and Taipei and rural areas Gender: 51.3% male Age: 15–24 years	Young people more likely to respect the filial piety to family and self-cultivation of virtues are less likely to have premarital sexual intercourse. Confucian culture promotes hugging, kissing or foundling to fulfill the sexual desires, as an alternative to vaginal sexual intercourse until marriage However, these constraints may keep secret the youth's sexual experiences to their parents, and evoke stigmatization and inhibit sexually active unmarried youth seeking reproductive health services
Garrison et al., 2018/ South Korea	To explore university Korean male students’ experiences related to their ability to attain prestigious hakbeol status	Qualitative (semistructured interview)	12 University Korean men attending of lesser-known universities Gender: 100% male Age: 19 – 26 years (M = 22.75)	Korean men with non-prestigious hakbeol reported psychological discomfort and experienced discrimination due to the societal expectations of filial piety placed on them
Holroyd, 2001/ China	To describe the caregiving obligations of Hong Kong Chinese daughters toward their elderly parents	Qualitative	20 caregiving daughters for their elderly parents Gender: 100% female Age: 18–65 years	For Hong Kong women caring for a parent, there was little need to emphasize filial commitment as a distinct duty. Caregiving obligation is driven by cumulated credit (reciprocity of gifts and services), contemporary government policy and strong emotions, rather than as a result of assertion of parental rights
Holroyd, 2003/ China	To understand how bodily order and disorder are managed in Chinese caregiving families	Qualitative (ethnographic research: interviews and participant observation over 2 year)	35 families caring for elderly, 9 families caring for chronically ill or disabled children, and 2 families caring for chronically ill or disabled adult children Gender: 68.57% female Age: Dependent children: 1–16 years Elderly dependent: 65–91 years	Based on the Confucian sense of order, families must regulate bodily illness (symbolic of disorder) beyond the filial piety understandings, to cover bodily needs (i.e., ingestion of food and medicine, excretions, washing, assisting with toileting, dressing), and to affirm their own social identity through social relationships with their dependents. This generates high levels of fatigue in caregivers from the incessant demands of meeting family duties and concern about death at home
Hsiao et al., 2006 / Australia	To understand the experiences of suffering, distress and emotional reactions of Chinese immigrant patients with mental illness, and the role of the family, within a cultural focus	Qualitative (phenomenological study with interviews)	28 Chinese patients and 28 caregivers living in Australia (total sample = 56) Gender: 54% of patients and 61% of caregivers were males Age: 45–46 years	Not fulfilling the cultural expectation of filial piety generates emotional distress, self-blame and shame because of the reproof from the parents. Destroying the harmony of the family and not fulfilling the responsibility and role within the family was recognized as a cause of family dishonor and depressive disorder
Jia & Zhang, 2017/ China	To explore the association between Confucian values and suicide with major depression in young rural Chinese victims	Quantitative (paired case–control psychological autopsy method)	392 consecutive suicide victims and 416 living controls Gender: 67.8% male Age: 15–34 years (M = 28.24)	Adherence to Confucian values (filial piety, harmony) and gender roles for men was negatively associated with suicide with major depression, but positively associated for women. Most negative life events (69.6%) involved interpersonal relationships (*renqing*), especially with family members
Jones & Nie, 2018 / China	To understand the popular views on Confucian attitudes toward body donation	Narrative review	–	Filial piety should not hamper body donation, but encourage it. It could be possible taking into account other Confucian cultural norms and rites, such as ren (humaneness or benevolence) and li (rites), that comprise the body donation as a communal activity to promote virtue cultivation
Kang & Crogan, 2008 / United States	To describe the social and cultural constructions that influence help-seeking for urinary incontinence (UI) among Korean American elderly women	Narrative review	–	Older women accept UI as a normal part of aging that should not be managed by health care providers, since UI is shameful, private and familiar and it is taken for granted that the family will care for those women (mainly daughters and daughters-in law) as a way of expressing filial piety and to avoid being blamed by their community
Lai & Surood, 2009/ Canada	To examine the nature and prevalence of culture- related health beliefs and values of aging Chinese immigrants in Canada	Quantitative (cross-sectional survey)	2272 older Chinese people in Canada Gender: 55,8% female Age: 55 years or older (*M* = 69.8)	Traditional health practice and the use of traditional Chinese medicine are based on Confucian concepts of harmony and being morally good. Body harmony implies seeking a balance between yin and yang, through traditional Chinese exercise and healthy diet A lower educational level and less exposure to Western culture and religion are related to greater adherence to traditional health practices
Laidlaw et al., 2010 / China and United Kingdom	To understand the expectations for filial support care from within the families among older people in Eastern and Western societies	Quantitative (Attitudes to ageing questionnaire-AAQ; Filial piety expectation questionnaire-FPEQ; Self-report measure of depression-CES-D)	130 older adults from different cultural groups: Scottish older people born in Edinburgh (*n* = 20) Chinese immigrants living in Scotland (*n* = 32) Chinese residents in Beijing (*n* = 78) Gender: 55% female Age: 60–92 years (*M* = 70.6)	The Chinese from China have a more negative attitude to aging in comparison with the two UK-based groups, relating it to physical changes and psychosocial loss. To compensate for the anticipated losses, the elderly have more expectations in the filial support of their children, and the greatest expectation of filial piety was related to the country of origin (China)
Lee et al., 2016 / China	To explore the perspectives of older nursing home residents in Taiwan toward signing their own “Do not Resuscitate” (DNR) directives	Qualitative (in-depth interviews and field notes)	11 residents from a nursing home Gender: 64% female Age: 65–92 years (M = 79 years)	Most residents refused to sign their DNR directives (n = 8; 72.7%), because: 1) they wanted to consult with their children, as the family is as a decision-making system where the filial behavior of children seek the best for their parents; 2) due to familism, they were concerned for causing problems and not showing sacrifice; 3) they considered death to be something natural and they did not want to go against nature
Li & Li, 2017 / China	To show Confucian views on human dignity and death and compare them with the Western points of view	Narrative review	–	Confucian ethics accepts death calmly as a law of nature, without lengthen the life deliberately through medical techniques. However, quality of life is equivalent to moral quality; therefore, when life contradicts morality or individuals cannot fulfill their events, they can preserve one's dignity by ending one's own life Universal dignity indicates that the family, the medical professional, and the entire society should all bear responsibility for taking care of the patient at the end of life. Filial piety can make an older person not decide autonomously to undergo euthanasia if the children object so as not to lose their own reputation. However, passive euthanasia does not threaten the children’s dignity
Lihua et al., 2017 / China	To explore self-compassion and Confucian coping to predict anxiety and depression in impoverished Chinese undergraduate students	Quantitative (Confucian Coping Scale; Self-Compassion Scale; Beck Depression Inventory; Beck Anxiety Inventory)	330 impoverished undergraduates Gender: 63% male Age: 16–24 years (*M* = 19.9)	Higher self-compassion, pro-setback thinking and responsibility thinking predicted lower depression and anxiety in impoverished undergraduates. However, fate thinking was related with higher depression and anxiety
Lo & Russell, 2007 / Australia	To research on the experience of family care’ among elderly Chinese immigrants in Australia	Qualitative (In‐depth interviews)	6 non-Australian-born Asian elderly people Gender: 83.3% female Age: 65–83 years	Although elderly Chinese immigrants expected their children's financial support for caring as a filial piety behavior, their health and welfare needs were not met through traditional family structures. Three participants with serious health problems who lived alone receiving most of their support from formal community services. This shows a tendency toward a 'westernisation' of elder care
Mayer et al., 2020 / Over the world (103 countries)	To determine the relationship between culture or level of social freedom and the management of pandemic challenges	Quantitative (country-level regression analysis)	–	Confucian and South Asian cultures were significantly better at preventing the spread of COVID-19 than the rest of the world, due to higher rates of obedience, great attention to cleanliness and non-physical greetings and farewells among people
Nie & Jones, 2019 / China	To analyze organ donation through the Confucian ethical outlook	Narrative review	–	There could be a discrepancy between filial piety and ren regarding the acceptance of organ donation. Confucian emphasis on filial piety requires body integrity after death as a signal of respect for one’s parents, although the complexity of applying it in medical settings is evidenced by considering this rule as a superficial reason (foolish filial piety or yuxiao) On the other hand, although donation of organs is an act of compassion (Confucian spirit of ren) working for the good of others, humanity and righteousness, Confucianism does not advocate loving a stranger as one would a family member, or having the same moral duty to every human being
Pang et al., 2015 / Australia	To examine Chinese migrant young people’s lifestyles and physical activity experiences regarding the values and cultural investments of their families in Australia	Qualitative, ethnography (interviews)	12 Chinese young people from two schools Gender: 83.3% female Age: 10–15 years	Parents conceptualized sport as valuable only if it contributed to substantial symbolic capital. Keeping social hierarchy of family by the intergenerational transmission of values implies for young people to devote themselves to academic and musical success, at the expense of school sport, to avoid humiliation and rejection by their parents. These Chinese family’s practices differed to the traditional Confucianism (e.g., "the unity of body and mind")
Park, 2012/ USA	To know whether Confucian practices of filial piety and parental obligation are present in modern Korean immigrant families in American society and describe their influence managing mental illness	Qualitative (in-depth, face-to-face interviews)	6 Korean Americans who have experienced caring for a mentally ill family member Gender: 100% female Age: 38–68 years (M = 52.8)	Different patterns of family coping were identified. Some parents managed their child's mental illness by limiting stressors (insulating from the outside world, including health providers support); others prioritized education over well-being so that he or she could be self-reliant. Families who place a mentally ill family member in an institution may feel guilty. Elderly Korean patients who are placed in a long-term care facility may feel ashamed because it implies that they failed to raise a good child who honors filial piety
Ran et al., 2021/ Asia, USA and Latin America areas	To know the cultural perception and influence on mental illnesses in the population of the Pacific Rim region	Systematic review	Forty-one studies in Pacific Rim region	Many Asians perceive persons with mental illness as representing the family’s mental illness and their bad past, and also mental illness is considered a strain on collective systems within Asia, including families or social groups. Therefore, this people might suffer stigmatizing and more physical threat. Avoiding disclosure of mental illness in order to save face in Confucian societies might lead to delayed treatment seeking
Raposo, 2019 / China	To describe the Confucian philosophical basis for patient informed consent	Narrative review	–	Confucianism influences in legal norms on informed consent and give both the patients and their relatives the same right to be informed and make decisions. Confucian model looks for a harmonious balance between the doctor, the family and the patient. However, in day-to-day practice, Confucian familism makes medical information to be offered to the family in the first instance, and if the family agrees, to the patient, filtering the information he/she receives. On many occasions, the informed consent form is signed only by one family member representing the entire family
Rochelle & Yim, 2014/ China	To explore patterns and factors associated with TCM use among Hong Kong Chinese	Quantitative (Chinese-Western Medical Beliefs Scale, Chinese Values Scale)	300 Hong Kong-born Chinese Gender: 71.7% female Age: 18–90 years (M = 42.1)	Belief in traditional Chinese values (respect for tradition, patriotism and sense of cultural superiority) was predictive of TCM utilization and of the belief in the effectiveness and superiority of TCM over WM. However, tolerance and harmony was not predictive of using TCM, and these participants took a more neutral point of view when comparing both models of medicine
Siu, 2003/ China	To explore the influence of the Chinese work values and organizational commitment on the stress–performance relationship	Quantitative (self-administered survey)	Employees in the service sector First sample: 389 Second sample: 145 Gender: 52.4% female Mean age: First sample: M = 38.31 years Second sample: M = 36.77	The traditional work-related Confucian values of Hong Kong employees (hardworking, endurance, reciprocity, and loyalty) were moderators of the stress–performance relationship, but only when stress was low or moderately high
Small & Blanc, 2021 / Vietnam	To examine the role of Tam Giao in resiliency in the face of COVID-19 in Vietnam	Narrative review	–	Based on Tam Giao, some behaviors to maintain social harmony are considered legal (including government responses), such as lying about the seriousness of COVID-19 to keep calm. On the other hand, Tam Giao also implies promoting community solidarity instead of individualist solutions (i.e., social distancing as a form of good karma, care toward elder populations or community policing), which prevents further spread of COVID-19
Tai & Tsai, 2003 / China	To investigate who makes decisions for a family member receiving diagnosis and medical treatment for cancer	Quantitative with survey	186 Taiwan people awaiting treatments at the Hospital (randomly selected) Age: 20–55 years	More so two-thirds of the participants said it should be revealed diagnosis of cancer to the head of the family. Concerning the course of the treatment, 57.1% indicated that the decision should be made either by the father or the husband of the patient (fulfilling their role and position in the family), and 7.4% stated that decision on a major operation should be made by the patient Filial piety implies respect and gratitude toward parents, so when parents become senile, usually the oldest son, assume the duty to take care of them, including making decisions
Tao et al., 2014 / China	To explore the self‐care behavior among Chinese adults with a permanent colostomy	Qualitative (In‐depth interviews)	7 Chinese adults at a hospital in southwest China Gender: 57% male Age: 39–68 years (*M* = 54)	Good care emphasizes more about individuals' efforts than good outcomes managing colostomy. On the one hand, the participants showed independence using stoma appliances and handling peristomal skin problem so that they do not feel themselves not being a burden to family, and to regain the power to repay their families. On the other hand, they were pleased that their families occasionally changed their stoma appliances expressing concern and love
Yang et al., 2016/ China	To examine the relationship and impact of Zhongyong thinking on mental health indicators in a representative nationwide sample in China	Quantitative (Two studies: National Mental Health Survey and clinical trial-Zhongyong thinking training)	National mental health survey: 8,341 students Gender: 61.3% female Age: 16–27 years (*M* = 21.1) Clinical trial: Total sample of 60 mildly depressed undergraduate students IG: *n* = 30 Gender: 80% female Mean age: 18.8 years CG: *n* = 30 Gender: 73.3% female Mean age: *M* = 18.6 years	Significant negative relationships between Zhongyong thinking and mental distress indicators, and positive relationships between Zhongyong thinking and subjective well-being indicators were found. In addition, study 2 demonstrated that depressive symptoms improved by training Zhongyong thinking in group therapy
Yiu et al., 2021 / China	To explore the influence of Confucianism on the process of caring among the Chinese family caregivers	Qualitative (Semistructured interviews)	15 Chinese family caregivers of persons with dementia in elderly care centers in Hong Kong Gender: 80% female Age: 74–89 years (*M* = 80.9)	In Confucianism, Chinese emphasize familism and the family unit as the primary social unit, so Chinese take care of their relative with Alzheimer's and sacrifice for him / her (including employment and leisure time) due to everything that this person did for them in the past Acceptance of the role as family caregivers is also related to the Confucian concept of Tian (heaven) Ming (destiny). In addition, within the family there are also relationships emphasized in Confucianism: father and son, brother and brother, and wife and husband, which makes them accept care as part of the fulfillment of roles in these family social relationships
Zhang, 2010/ China	To discuss the concept of community from a Confucian perspective and its relevance for the current public healthcare in China	Narrative review	–	The children of a Confucian family are obliged to fulfill the moral duty, reverence and care of their parents. However, Confucianism also implies helping those in need, so it is not totally opposed to the government assuming responsibility to provide public healthcare to those who cannot afford to pay for basic healthcare needs
Zhang et al., 2020/ China	To assess the perception and attitude toward body donation	Quantitative with survey	2535 community residents in Changsha Gender: 56.4% female Age: 18–89 years (*M* = 39.4)	There is a correlation between Confucianism funeral belief (when a person dies, the body must be returned in the original form) and the willingness to donate one's body (p < 0.001). Participants with no Confucianism funeral belief were 9.8 times more willing to donate their bodies

3
